# 2-(4-Meth­oxy-2-methyl­anilino)-1,2-diphenyl­ethanone

**DOI:** 10.1107/S1600536811015960

**Published:** 2011-05-07

**Authors:** Hakan Arslan, Oztekin Algul, Selin Zirek, Ozden Tari, Aydın Demircan, Kenneth I. Hardcastle

**Affiliations:** a Department of Chemistry, Emory University, Atlanta, GA 30322, USA; b Department of Chemistry, Faculty of Arts and Science, Mersin University, Mersin, TR 33343, Turkey; c Department of Pharmaceutical Chemistry, Faculty of Pharmacy, Mersin University, Mersin, TR 33169, Turkey; d Department of Chemistry, Faculty of Arts and Sciences, Nigde University, Nigde, TR 51240, Turkey

## Abstract

The title compound, C_22_H_21_NO_2_, was synthesized from 4-meth­oxy-2-methyl­aniline and 2-hy­droxy-1,2-diphenyl­ethanone. In the title compound, the C—C—C—N—C backbone adopts an all-*trans* conformation. The crystal structure is stabilized by weak inter­molecular C—H⋯O hydrogen-bond inter­actions.

## Related literature

For the synthesis and similar structures, see: Au & Tafeenko (1986 ▶, 1987 ▶); Batsanov *et al.*, (2006 ▶). For general background to these structures, see: Batsanov *et al.* (2006 ▶); Abdulla *et al.* (1985 ▶). For bond-length data, see: Allen *et al.* (1987 ▶). For geometrical analysis, see: Bruno *et al.* (2002 ▶).
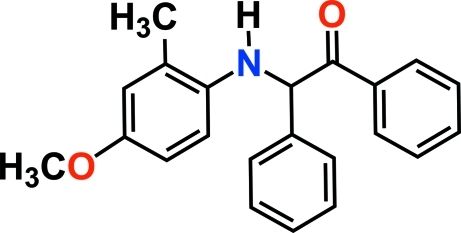


## Experimental

### 

#### Crystal data

C_22_H_21_NO_2_
*M* *_r_* = 331.40
Monoclinic, 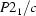

*a* = 12.570 (12) Å
*b* = 8.009 (8) Å
*c* = 18.091 (17) Å
β = 100.544 (15)°
*V* = 1791 (3) Å^3^
*Z* = 4
Mo *K*α radiation
μ = 0.08 mm^−1^
*T* = 173 K
0.21 × 0.19 × 0.15 mm

#### Data collection

Bruker SMART CCD area-detector diffractometer
Absorption correction: multi-scan (*SADABS*; Bruker, 2008 ▶) *T*_min_ = 0.984, *T*_max_ = 0.988
28799 measured reflections
4122 independent reflections
2935 reflections with *I* > 2σ(*I*)
*R*_int_ = 0.055

#### Refinement

*R*[*F* ^2^ > 2σ(*F* ^2^)] = 0.042
*wR*(*F* ^2^) = 0.112
*S* = 1.05
4122 reflections
226 parameters
H-atom parameters constrained
Δρ_max_ = 0.27 e Å^−3^
Δρ_min_ = −0.25 e Å^−3^

### 

Data collection: *SMART* (Bruker, 2008 ▶); cell refinement: *SAINT* (Bruker, 2008 ▶); data reduction: *SAINT*; program(s) used to solve structure: *SHELXS97* (Sheldrick, 2008 ▶); program(s) used to refine structure: *SHELXL97* (Sheldrick, 2008 ▶); molecular graphics: *OLEX2* (Dolomanov *et al.*, 2009 ▶); software used to prepare material for publication: *SHELXTL* (Sheldrick, 2008 ▶), *OLEX2*, *publCIF* (Westrip, 2010 ▶) and *Mercury* (Macrae *et al.*, 2006 ▶).

## Supplementary Material

Crystal structure: contains datablocks I, global. DOI: 10.1107/S1600536811015960/hg5030sup1.cif

Structure factors: contains datablocks I. DOI: 10.1107/S1600536811015960/hg5030Isup2.hkl

Supplementary material file. DOI: 10.1107/S1600536811015960/hg5030Isup3.cml

Additional supplementary materials:  crystallographic information; 3D view; checkCIF report

## Figures and Tables

**Table 1:** Hydrogen-bond geometry (Å, °)

*D*—H⋯*A*	*D*—H	H⋯*A*	*D*⋯*A*	*D*—H⋯*A*
C11—H11*A*⋯O1^i^	0.95	2.48	3.352 (4)	153

Symmetry code: (i) 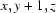.

**Fig. 1.**
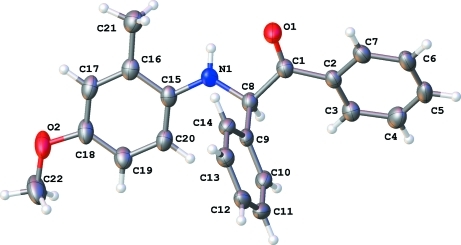
The molecular structure of (I), showing ellipsoids at the 50% probability level.

**Fig. 2.**
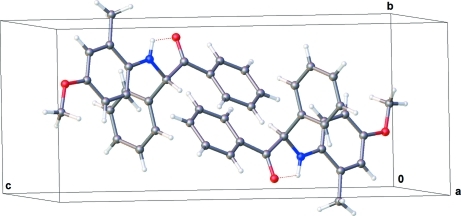
The molecular packing of (I). The hydrogen bonds are shown as dashed lines.

**Fig. 3.**
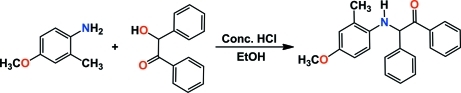
Synthesis of the title compound.

## supplementary crystallographic information

### Comment

A few 1-arylanilinoethanone derivatives have been structurally charactized because of their importance in synthesis or because of their interesting charge-transfer properties (Abdulla *et al.*, 1985). We found only four structurally similar compounds (Au & Tafeenko, 1987; Au & Tafeenko, 1986; Batsanov *et al.*, 2006) in the Cambridge Structural Database (CSD CONQUEST 1.11, B4; Bruno *et al.*, 2002).

Compound I was synthesized by the HCl acid-catalyzed reaction of a benzoin derivative to 4-methoxy-2-methylaniline (Scheme 1) resulting in the title compound, 2-((4-methoxy-2-methylphenyl)amino)-1,2-diphenylethanone, I, Figure 1, its structure was determined by X-ray crystallography.

The molecular structure of the title compound contains two phenyl rings and one substituted aniline ring connected by a –C(O)—C– linker. The dihedral angle between the two phenyl rings is 87.78 (7) ° and the dihedral angle between the substitute aniline and phenyl rings are 55.30 (7) and 84.57 (7) °, respectively. In adition, in the title compound, the C2—C1—C8—N1—C15 backbone adopts an all-*trans* conformation (Au & Tafeenko, 1987; Au & Tafeenko, 1986; Batsanov *et al.*, 2006).

The C—N bond lengths C8—N1 and C15—N1 are shorter than the normal C—N single-bond length of about 1.48 Å. The shortening of these C—N bonds reveals the effects of some conjugation in this part of the molecule. All other bond lengths fall within the expected ranges (Allen *et al.*, 1987).

Intramolecular hydrogen bonding N1–H1A···O1 with N–H 0.88 Å, H···O 2.22 Å, N–H···O 107 ° results in the formation of a five membered ring in the O1—C1—C8—N1–H1A plane. The crystal packing is dominated by weak intermolecular C11—H11A···O1 (*x*, 1 + *y*, *z*) hydrogen bonds, with H···O = 2.48 Å and a C—H···O angle of 153 ° (Figure 2).

### Experimental

A mixture of 4-methoxy-2-methylaniline (15 mmol), 2-hydroxy-1,2-diphenylethanone (5 mmol) and 1 ml conc. HCl in 20 ml of ethanol were refluxed for 5 h (Figure 3). After reaction was complete, the mixture was allowed to cool to room temperature, poured into cold water (20 ml) and finally extracted with CH_2_Cl_2_ (3x15 ml). The organic layer was dried over magnesium sulfate and the solvent removed under reduced pressure to yield a crude product that was purified by recrystallization in ethyl acetate. 2-((4-methoxy-2-methylphenyl)amino)-1,2-diphenylethanone: Yield: 1.20 g, 46%. *M*.p.: 110–112 °C. ^1^H NMR (DMSO-*d*_6_) δ: 8.18–8.16 (d, 2H, Ar—H (C3, C7)), 7.62 (t, 1H, Ar—H (C5)), 7.56–7.49 (m, 4H, Ar—H (C4, C6, C11, C13)), 7.30–7.26 (m, 2H, Ar—H (C10, C14)), 7.16 (t, 1H, Ar—H (C12)), 6.70–6.68 (d, *J*=4.8 Hz, 1H, Ar—H (C19)), 6.67 (s, 1H, Ar—H (C17)), 6.57–6.55 (d, *J*=8 Hz, 1H, Ar—H (C20)), 6.46–6.44 (d, *J*=8 Hz, 1H, Ar—H (C8)), 5.19–5.17 (d, *J*=8 Hz, 1H, NH), 3.60 (s, 3H, OCH_3_), 2.22 (s, 3H, CH_3_). ^13^C NMR (400 MHz, p.p.m.) δ: 17.5 (CH_3_), 55.1 (OCH_3_), 61.5 (C8), 111.2 (C19), 112.5 (C20), 116.4 (C17), 123.9 (C16), 127.6, 128.1, 128.6, 128.7, 128.8 (C), 133.7 (C5), 134.7 (C2), 138.1 (C9), 138.3 (C15), 151.1 (C18), 197.6 (C9). Anal. Calc. for C_22_H_21_NO_2_: C, 79.73; H, 6.39; N, 4.23%. Found: C, 79.70; H, 6.21; N, 4.19%.

### Refinement

H atom positions were clearly derived from difference Fourier maps and refined using a riding model, fixing the bond lengths at 0.98 and 0.95 Å for CH_3_ and CH(aromatic), respectively. The displacement parameters of the H atoms were constrained with *U*_iso_(H) = 1.2*U*_eq_ (C) or 1.5*U*_eq_ (methyl C).

### Crystal data

**Table:**

C_22_H_21_NO_2_	*F*(000) = 704
*M**_r_* = 331.40	*D*_x_ = 1.229 Mg m^−^^3^
Monoclinic, *P*2_1_/*c*	Mo *K*α radiation, λ = 0.71073 Å
Hall symbol: -P 2ybc	Cell parameters from 4852 reflections
*a* = 12.570 (12) Å	θ = 2.3–24.5°
*b* = 8.009 (8) Å	µ = 0.08 mm^−^^1^
*c* = 18.091 (17) Å	*T* = 173 K
β = 100.544 (15)°	Block, yellow
*V* = 1791 (3) Å^3^	0.21 × 0.19 × 0.15 mm
*Z* = 4	

### Data collection

**Table:**

Bruker SMART CCD area-detector diffractometer	4122 independent reflections
Radiation source: fine-focus sealed tube	2935 reflections with *I* > 2σ(*I*)
graphite	*R*_int_ = 0.055
φ and ω scans	θ_max_ = 27.5°, θ_min_ = 1.7°
Absorption correction: multi-scan (*SADABS*; Bruker, 2008)	*h* = −16→16
*T*_min_ = 0.984, *T*_max_ = 0.988	*k* = −10→10
28799 measured reflections	*l* = −23→23

### Refinement

**Table:**

Refinement on *F*^2^	Primary atom site location: structure-invariant direct methods
Least-squares matrix: full	Secondary atom site location: difference Fourier map
*R*[*F*^2^ > 2σ(*F*^2^)] = 0.042	Hydrogen site location: inferred from neighbouring sites
*wR*(*F*^2^) = 0.112	H-atom parameters constrained
*S* = 1.05	*w* = 1/[σ^2^(*F*_o_^2^) + (0.0413*P*)^2^ + 0.4229*P*] where *P* = (*F*_o_^2^ + 2*F*_c_^2^)/3
4122 reflections	(Δ/σ)_max_ = 0.004
226 parameters	Δρ_max_ = 0.27 e Å^−^^3^
0 restraints	Δρ_min_ = −0.25 e Å^−^^3^

### Special details

**Table:**

Geometry. All e.s.d.'s (except the e.s.d. in the dihedral angle between two l.s. planes) are estimated using the full covariance matrix. The cell e.s.d.'s are taken into account individually in the estimation of e.s.d.'s in distances, angles and torsion angles; correlations between e.s.d.'s in cell parameters are only used when they are defined by crystal symmetry. An approximate (isotropic) treatment of cell e.s.d.'s is used for estimating e.s.d.'s involving l.s. planes.
Refinement. Refinement of *F*^2^ against ALL reflections. The weighted *R*-factor *wR* and goodness of fit *S* are based on *F*^2^, conventional *R*-factors *R* are based on *F*, with *F* set to zero for negative *F*^2^. The threshold expression of *F*^2^ > σ(*F*^2^) is used only for calculating *R*-factors(gt) *etc*. and is not relevant to the choice of reflections for refinement. *R*-factors based on *F*^2^ are statistically about twice as large as those based on *F*, and *R*- factors based on ALL data will be even larger.

### Fractional atomic coordinates and isotropic or equivalent isotropic displacement parameters (Å^2^)

**Table:**

	*x*	*y*	*z*	*U*_iso_*/*U*_eq_	
C1	0.70413 (11)	0.71666 (18)	0.13255 (8)	0.0336 (3)	
C2	0.65511 (11)	0.79413 (17)	0.05905 (7)	0.0314 (3)	
C3	0.70228 (12)	0.92642 (19)	0.02686 (8)	0.0380 (3)	
H3A	0.7667	0.9764	0.0533	0.046*	
C4	0.65510 (14)	0.9856 (2)	−0.04399 (8)	0.0453 (4)	
H4A	0.6879	1.0748	−0.0662	0.054*	
C5	0.56034 (14)	0.9144 (2)	−0.08206 (8)	0.0436 (4)	
H5A	0.5286	0.9546	−0.1305	0.052*	
C6	0.51179 (13)	0.7853 (2)	−0.05009 (8)	0.0420 (4)	
H6A	0.4460	0.7385	−0.0761	0.050*	
C7	0.55897 (12)	0.72392 (18)	0.01989 (8)	0.0361 (3)	
H7A	0.5260	0.6339	0.0414	0.043*	
C8	0.78651 (11)	0.81396 (18)	0.19059 (8)	0.0339 (3)	
H8A	0.8385	0.8744	0.1644	0.041*	
C9	0.72052 (11)	0.94044 (17)	0.22728 (7)	0.0297 (3)	
C10	0.72302 (12)	1.11045 (18)	0.21167 (8)	0.0370 (3)	
H10A	0.7685	1.1508	0.1790	0.044*	
C11	0.65930 (13)	1.22179 (18)	0.24361 (9)	0.0421 (4)	
H11A	0.6615	1.3376	0.2327	0.051*	
C12	0.59250 (12)	1.16418 (19)	0.29135 (8)	0.0396 (4)	
H12A	0.5489	1.2403	0.3129	0.048*	
C13	0.58976 (12)	0.99566 (19)	0.30736 (8)	0.0378 (3)	
H13A	0.5441	0.9557	0.3399	0.045*	
C14	0.65372 (11)	0.88458 (18)	0.27584 (8)	0.0334 (3)	
H14A	0.6519	0.7691	0.2875	0.040*	
C15	0.91952 (11)	0.72896 (19)	0.30593 (8)	0.0330 (3)	
C16	0.95263 (11)	0.59902 (19)	0.35825 (8)	0.0346 (3)	
C17	1.02943 (11)	0.6351 (2)	0.42183 (8)	0.0393 (4)	
H17A	1.0524	0.5485	0.4570	0.047*	
C18	1.07393 (12)	0.7936 (2)	0.43580 (8)	0.0412 (4)	
C19	1.04216 (12)	0.9204 (2)	0.38448 (8)	0.0409 (4)	
H19A	1.0725	1.0289	0.3929	0.049*	
C20	0.96492 (11)	0.88712 (19)	0.32004 (8)	0.0375 (3)	
H20A	0.9429	0.9745	0.2851	0.045*	
C21	0.90500 (13)	0.4262 (2)	0.34587 (9)	0.0442 (4)	
H21A	0.9374	0.3535	0.3875	0.066*	
H21B	0.9201	0.3810	0.2985	0.066*	
H21C	0.8266	0.4318	0.3435	0.066*	
C22	1.19345 (16)	0.9696 (3)	0.52047 (11)	0.0690 (6)	
H22A	1.2440	0.9640	0.5686	0.103*	
H22B	1.1358	1.0496	0.5244	0.103*	
H22C	1.2322	1.0058	0.4810	0.103*	
N1	0.84386 (10)	0.69119 (16)	0.24129 (7)	0.0412 (3)	
H1A	0.8302	0.5852	0.2308	0.049*	
O1	0.67637 (9)	0.57676 (13)	0.14827 (6)	0.0477 (3)	
O2	1.14758 (10)	0.80923 (17)	0.50230 (6)	0.0612 (4)	

### Atomic displacement parameters (Å^2^)

**Table:**

	*U*^11^	*U*^22^	*U*^33^	*U*^12^	*U*^13^	*U*^23^
C1	0.0331 (7)	0.0372 (8)	0.0294 (7)	−0.0008 (6)	0.0026 (6)	−0.0002 (6)
C2	0.0323 (7)	0.0345 (8)	0.0264 (7)	0.0029 (6)	0.0024 (6)	−0.0041 (6)
C3	0.0388 (8)	0.0420 (8)	0.0318 (8)	−0.0004 (7)	0.0030 (6)	−0.0009 (6)
C4	0.0585 (10)	0.0449 (9)	0.0338 (8)	0.0049 (8)	0.0114 (7)	0.0045 (7)
C5	0.0558 (10)	0.0458 (9)	0.0256 (7)	0.0141 (8)	−0.0022 (7)	−0.0025 (6)
C6	0.0435 (9)	0.0450 (9)	0.0326 (8)	0.0067 (7)	−0.0062 (7)	−0.0089 (7)
C7	0.0386 (8)	0.0360 (8)	0.0318 (7)	0.0014 (6)	0.0013 (6)	−0.0056 (6)
C8	0.0314 (7)	0.0377 (8)	0.0299 (7)	−0.0001 (6)	−0.0013 (6)	0.0027 (6)
C9	0.0279 (7)	0.0321 (7)	0.0252 (7)	−0.0033 (6)	−0.0055 (5)	0.0023 (5)
C10	0.0399 (8)	0.0347 (8)	0.0331 (7)	−0.0082 (6)	−0.0024 (6)	0.0067 (6)
C11	0.0503 (9)	0.0265 (7)	0.0418 (8)	−0.0024 (7)	−0.0121 (7)	0.0012 (6)
C12	0.0401 (8)	0.0371 (8)	0.0362 (8)	0.0056 (7)	−0.0076 (7)	−0.0060 (6)
C13	0.0381 (8)	0.0399 (8)	0.0337 (7)	−0.0025 (7)	0.0023 (6)	−0.0008 (6)
C14	0.0373 (8)	0.0288 (7)	0.0314 (7)	−0.0032 (6)	−0.0006 (6)	0.0029 (6)
C15	0.0260 (7)	0.0444 (8)	0.0281 (7)	0.0042 (6)	0.0033 (5)	0.0017 (6)
C16	0.0279 (7)	0.0453 (9)	0.0315 (7)	0.0023 (6)	0.0084 (6)	0.0050 (6)
C17	0.0302 (7)	0.0542 (10)	0.0333 (8)	0.0001 (7)	0.0047 (6)	0.0151 (7)
C18	0.0297 (8)	0.0616 (10)	0.0295 (7)	−0.0071 (7)	−0.0016 (6)	0.0101 (7)
C19	0.0351 (8)	0.0499 (9)	0.0356 (8)	−0.0089 (7)	0.0011 (6)	0.0052 (7)
C20	0.0337 (8)	0.0445 (9)	0.0323 (7)	0.0018 (7)	0.0007 (6)	0.0082 (6)
C21	0.0437 (9)	0.0476 (9)	0.0407 (8)	−0.0011 (7)	0.0062 (7)	0.0079 (7)
C22	0.0614 (12)	0.0892 (15)	0.0463 (10)	−0.0308 (11)	−0.0170 (9)	0.0086 (10)
N1	0.0441 (7)	0.0359 (7)	0.0371 (7)	0.0081 (6)	−0.0094 (6)	−0.0035 (5)
O1	0.0572 (7)	0.0424 (6)	0.0380 (6)	−0.0124 (5)	−0.0057 (5)	0.0068 (5)
O2	0.0565 (7)	0.0759 (9)	0.0411 (6)	−0.0243 (7)	−0.0179 (6)	0.0195 (6)

### Geometric parameters (Å, °)

**Table:**

C1—O1	1.2226 (19)	C12—H12A	0.9500
C1—C2	1.494 (2)	C13—C14	1.390 (2)
C1—C8	1.544 (2)	C13—H13A	0.9500
C2—C3	1.393 (2)	C14—H14A	0.9500
C2—C7	1.402 (2)	C15—C20	1.394 (2)
C3—C4	1.393 (2)	C15—N1	1.399 (2)
C3—H3A	0.9500	C15—C16	1.417 (2)
C4—C5	1.385 (2)	C16—C17	1.390 (2)
C4—H4A	0.9500	C16—C21	1.509 (2)
C5—C6	1.380 (2)	C17—C18	1.391 (2)
C5—H5A	0.9500	C17—H17A	0.9500
C6—C7	1.386 (2)	C18—O2	1.383 (2)
C6—H6A	0.9500	C18—C19	1.385 (2)
C7—H7A	0.9500	C19—C20	1.399 (2)
C8—N1	1.4443 (19)	C19—H19A	0.9500
C8—C9	1.535 (2)	C20—H20A	0.9500
C8—H8A	1.0000	C21—H21A	0.9800
C9—C10	1.392 (2)	C21—H21B	0.9800
C9—C14	1.395 (2)	C21—H21C	0.9800
C10—C11	1.393 (2)	C22—O2	1.422 (2)
C10—H10A	0.9500	C22—H22A	0.9800
C11—C12	1.389 (2)	C22—H22B	0.9800
C11—H11A	0.9500	C22—H22C	0.9800
C12—C13	1.382 (2)	N1—H1A	0.8800
			
O1—C1—C2	119.92 (13)	C12—C13—H13A	120.0
O1—C1—C8	119.27 (13)	C14—C13—H13A	120.0
C2—C1—C8	120.77 (13)	C13—C14—C9	120.87 (14)
C3—C2—C7	119.18 (14)	C13—C14—H14A	119.6
C3—C2—C1	123.38 (13)	C9—C14—H14A	119.6
C7—C2—C1	117.39 (13)	C20—C15—N1	122.87 (13)
C2—C3—C4	120.09 (15)	C20—C15—C16	119.00 (14)
C2—C3—H3A	120.0	N1—C15—C16	118.12 (14)
C4—C3—H3A	120.0	C17—C16—C15	118.33 (15)
C5—C4—C3	119.99 (16)	C17—C16—C21	120.70 (13)
C5—C4—H4A	120.0	C15—C16—C21	120.97 (14)
C3—C4—H4A	120.0	C16—C17—C18	122.38 (14)
C6—C5—C4	120.41 (15)	C16—C17—H17A	118.8
C6—C5—H5A	119.8	C18—C17—H17A	118.8
C4—C5—H5A	119.8	O2—C18—C19	125.58 (15)
C5—C6—C7	120.02 (15)	O2—C18—C17	115.06 (13)
C5—C6—H6A	120.0	C19—C18—C17	119.35 (14)
C7—C6—H6A	120.0	C18—C19—C20	119.34 (15)
C6—C7—C2	120.30 (15)	C18—C19—H19A	120.3
C6—C7—H7A	119.9	C20—C19—H19A	120.3
C2—C7—H7A	119.9	C15—C20—C19	121.61 (14)
N1—C8—C9	114.91 (13)	C15—C20—H20A	119.2
N1—C8—C1	106.44 (13)	C19—C20—H20A	119.2
C9—C8—C1	106.20 (12)	C16—C21—H21A	109.5
N1—C8—H8A	109.7	C16—C21—H21B	109.5
C9—C8—H8A	109.7	H21A—C21—H21B	109.5
C1—C8—H8A	109.7	C16—C21—H21C	109.5
C10—C9—C14	118.68 (13)	H21A—C21—H21C	109.5
C10—C9—C8	121.58 (13)	H21B—C21—H21C	109.5
C14—C9—C8	119.71 (13)	O2—C22—H22A	109.5
C9—C10—C11	120.40 (15)	O2—C22—H22B	109.5
C9—C10—H10A	119.8	H22A—C22—H22B	109.5
C11—C10—H10A	119.8	O2—C22—H22C	109.5
C12—C11—C10	120.28 (15)	H22A—C22—H22C	109.5
C12—C11—H11A	119.9	H22B—C22—H22C	109.5
C10—C11—H11A	119.9	C15—N1—C8	124.59 (13)
C13—C12—C11	119.71 (14)	C15—N1—H1A	117.7
C13—C12—H12A	120.1	C8—N1—H1A	117.7
C11—C12—H12A	120.1	C18—O2—C22	117.49 (13)
C12—C13—C14	120.05 (15)		
			
O1—C1—C2—C3	−160.36 (14)	C11—C12—C13—C14	0.1 (2)
C8—C1—C2—C3	22.1 (2)	C12—C13—C14—C9	−0.6 (2)
O1—C1—C2—C7	17.0 (2)	C10—C9—C14—C13	0.8 (2)
C8—C1—C2—C7	−160.60 (12)	C8—C9—C14—C13	−177.33 (12)
C7—C2—C3—C4	−1.2 (2)	C20—C15—C16—C17	0.0 (2)
C1—C2—C3—C4	176.15 (14)	N1—C15—C16—C17	178.73 (13)
C2—C3—C4—C5	0.9 (2)	C20—C15—C16—C21	179.37 (13)
C3—C4—C5—C6	0.3 (2)	N1—C15—C16—C21	−1.9 (2)
C4—C5—C6—C7	−1.3 (2)	C15—C16—C17—C18	0.4 (2)
C5—C6—C7—C2	1.0 (2)	C21—C16—C17—C18	−178.96 (14)
C3—C2—C7—C6	0.2 (2)	C16—C17—C18—O2	178.75 (13)
C1—C2—C7—C6	−177.25 (13)	C16—C17—C18—C19	−0.8 (2)
O1—C1—C8—N1	21.30 (18)	O2—C18—C19—C20	−178.70 (14)
C2—C1—C8—N1	−161.10 (12)	C17—C18—C19—C20	0.8 (2)
O1—C1—C8—C9	−101.58 (16)	N1—C15—C20—C19	−178.65 (14)
C2—C1—C8—C9	76.02 (16)	C16—C15—C20—C19	0.0 (2)
N1—C8—C9—C10	135.07 (14)	C18—C19—C20—C15	−0.4 (2)
C1—C8—C9—C10	−107.56 (15)	C20—C15—N1—C8	−14.1 (2)
N1—C8—C9—C14	−46.83 (17)	C16—C15—N1—C8	167.24 (13)
C1—C8—C9—C14	70.54 (15)	C9—C8—N1—C15	−57.18 (19)
C14—C9—C10—C11	−0.5 (2)	C1—C8—N1—C15	−174.41 (13)
C8—C9—C10—C11	177.64 (12)	C19—C18—O2—C22	1.2 (2)
C9—C10—C11—C12	−0.1 (2)	C17—C18—O2—C22	−178.33 (16)
C10—C11—C12—C13	0.3 (2)		

### Hydrogen-bond geometry (Å, °)

**Table:**

*D*—H···*A*	*D*—H	H···*A*	*D*···*A*	*D*—H···*A*
N1—H1A···O1	0.88	2.22	2.609 (3)	107
C11—H11A···O1^i^	0.95	2.48	3.352 (4)	153

Symmetry codes: (i) *x*, *y*+1, *z*.
